# Immunotherapy and Systemic Treatment of Cutaneous Squamous Cell Carcinoma

**DOI:** 10.5826/dpc.11S2a169S

**Published:** 2021-10-01

**Authors:** Nader Aboul-Fettouh, Daniel Morse, Jigar Patel, Michael R. Migden

**Affiliations:** 1 Department of Dermatology, The University of Texas McGovern Medical School at Houston, Houston, TX, USA; 2 Department of Dermatology, The University of Texas MD Anderson Cancer Center, Houston, TX, USA; 3 Departments of Dermatology and Head and Neck Surgery, The University of Texas MD Anderson Cancer Center, Houston, TX, USA

**Keywords:** cutaneous squamous cell carcinoma, systemic therapies, immunotherapy, tumor response, cSCC, advanced cSCC

## Abstract

Cutaneous squamous cell carcinomas (cSCC) represent one of the most diagnosed non-melanoma skin cancers and its incidence is increasing globally. Whereas early stage and low risk cSCC is typically treated with surgery, and in some cases other localized therapeutic modalities, locally advanced or metastatic cSCC is a cause of significant morbidity and mortality that requires a different approach to therapy. Therapeutic attempts at treating advanced cSCC include a multi-disciplinary approach with considerations for surgery, radiation, and systemic therapies. In this review, we will discuss the various systemic therapies that have been trialed for advanced cSCC, beginning with the early cytotoxic and platinum-based agents as well as their corresponding limitations. We will then review the targeted approaches using EGFR inhibitors prior to discussing the more recent immunotherapeutics that have shown good tumor responses in this often-lethal disease.

## Introduction

The increasing global incidence of cutaneous squamous cell carcinoma (cSCC) is an emerging health crisis [1, 2]. Studies in the United Kingdom, Sweden, and Germany suggest an incidence of cSCC between 9–96 per 100,000 males and 5–68 per 100,000 females [3–5], where Australian data reveals an even higher incidence of 499 per 100,000 males and 291 per 100,000 females [6]. Variations in cSCC incidence may be due to differing nosologic classifications of cSCC between regions, which may include actinic keratoses or be limited to only invasive disease. A population-based study by the Mayo Clinic revealed that between 1976–1984 and 2000–2010 the incidence of cSCC increased by 263% [7]. In addition, recent reports show an increasing incidence of SCC relative to BCC in the aging population [8].

The majority of early stage cSCC cases are successfully treated by surgery. In some cases, other localized therapeutic modalities may be used [9,10]. These include topical cytostatic therapy (5-fluorouracil), topical immunotherapy (imiquimod), or intralesional injection (methotrexate, bleomycin). Destructive methods with cryosurgery, lasers, photodynamic therapy, radiotherapy, or curettage and electrodessication may also be used in the appropriate clinical context.

Approximately 5% of cSCC progress into advanced cSCC [11]. Advanced cSCC is comprised of locally advanced contiguous disease that is a poor candidate for surgery, single-modality radiation, or a combination of the two; and metastatic cSCC. In cases of larger, more aggressive cSCC where surgery can feasibly remove all of the tumor burden, it may come with the cost of significant morbidity and/or disfigurement from damage to nearby anatomical structures and thus is not a reasonable option. Treatment of metastatic cSCC also presents major challenges. Surgery, radiotherapy, and systemic therapy may be used in an attempt to control disease. Surgery can be used for tumor debulking as well as excision of isolated metastatic lesions or involved lymph nodes. Radiotherapy may be beneficial in treating bone metastases and locally advanced symptomatic tumors. In certain circumstances, these modalities can be used with a palliative intent in order to improve a patient’s quality of life. Combining surgery and radiotherapy with systemic therapy can be helpful, as complete remission of disease with purely systemic therapy is not guaranteed.

Until recently, systemic treatment for cSCC was not highly effective and often difficult to tolerate, thus negatively impacting patient’s quality of life. In this article, we will review the various systemic agents that have been used to treat advanced cSCC. Our discussion will range from the older, more antiquated, therapies, to modern small molecule targeted therapies and immunotherapies, of which PD-1 inhibitors have shown adequate tumor responses for locally advanced or metastatic cSCC. We will re-examine the systemic agents that have been trialed for advanced cSCC, discuss the limitations of each agent, and highlight future considerations and investigations needed to optimize systemic treatment for advanced cSCC.

### Cytotoxic Chemotherapy for cSCC

Prior to the development of more modern systemic agents used today, cytotoxic chemotherapy and platinum-based agents were the mainstay of therapy for advanced cSCC. Numerous agents, including cisplatin, bleomycin, interferon, 5-fluorouracil and doxorubicin had been trialed; many were published as case reports or case series. These articles reported widely varying response rates from 17%–84% [12–14]. Very few adequately powered randomized controlled trials were available to guide treatment regimens and there were no formal treatment recommendations. In many cases, these agents were combined with other therapy modalities such as radiotherapy or surgery which further confounded long term data evaluation of these treatment regimens [15]. Results showed short progression free survival (PFS) and/or overall survival (OS). In several cases reporting long-term tumor remission, patients had received subsequent surgical or radiation therapy. Table 1 summarizes several studies investigating cytotoxic agents for treatment of advanced cSCC and their limitations. These therapies are frequent causes of nausea and emesis, and at times severe hematological toxicities including neutropenia, thrombocytopenia, and anemia [16]. The use of cisplatin in combination with 5-FU and bleomycin showed an objective response rate (ORR) of 84% [13] but these types of regimens are not commonly used due to the significant toxicity that is not tolerable in the elderly population which makes up a large portion of the cSCC population. In this small combination study, the median age of the 14 patients enrolled was 59, patients had only locally developed tumors (none with metastasis), and 10 patients received adjuvant surgery or radiotherapy. It is worthwhile to note that these therapies continue to be under investigation in combination with more modern small molecule targeted therapies and immunotherapies that are the mainstay of our discussion in this article [17].

## Targeted Therapy for Advanced cSCC

Epidermal growth factor receptor (EGFR) is a member of the ErbB family of tyrosine kinase (RTKs) receptors and transmits a growth-inducing signal to cells when stimulated by an EGFR ligand [18]. EGFR is expressed in multiple organs and plays important roles in proliferation, survival, and differentiation of cells in both physiology and tumor development [19]. The activation of EGFR affects numerous biochemical pathways including RAS-RAF-MEK-MAPK, PLC-gamma/PKC and PI3K/AKT/mTOR, STAT and NFkB [20]. These pathways, frequently altered in cSCC, allow for increased tumor cell proliferation, migration, survival, altered differentiation, and resistance to apoptosis [21]. Differences in expression of EGFR between primary and metastatic lesions of cSCC have been reported, although EGFR expression is not always a requirement for cSCC carcinogenesis [22]. EGFR inhibitors were one of the earliest systemic targeted therapies for the treatment of advanced cSCC. Both monoclonal antibodies against the ligand binding domain of EGFR, as well as small tyrosine kinase inhibitors (TKI)molecules, have been trialed.

Cetuximab, a human/mouse chimeric monoclonal antibody, competitively binds and inhibits the ligand binding domain of EGFR thus limiting its interaction with the EGFR-ligand. A phase II study of cetuximab as first-line single-drug therapy in patients with advanced cSCC, conducted by Maubec and colleagues [23], included 36 patients that received cetuximab at an initial dose of 400 mg/m^2^ followed by subsequent weekly doses of 250 mg/m^2^ for at least 6 weeks with a 48 week follow up. ORR was 28%, with 2 complete remissions (6%) and 8 partial responses (22%), and a short mean OS of 8.1 months. Notably, the safety profile of the study was acceptable and similar to that of other studies. A larger retrospective clinical trial by Montaudié and colleagues, showed an ORR of 42% and 70% disease control rate (DCR) at 12 weeks in chemotherapy-naïve patients treated with cetuximab for advanced cSCC. Median OS was 17.5 months, and the majority of adverse events were grade 1 to 2. Notably, this trial included a large proportion of immunocompromised patients (~33% of the 58-patient cohort) which are typically excluded from clinical trials. The authors did note that their trial included a significantly higher portion of patients with only local disease (~66%) compared to that of the Maubec study (39%). This same group had also reported an ORR of 48% and DCR of 68% at six weeks in a separate retrospective cohort of 31 patients treated with cetuximab, with 47% of patients having local disease rather than metastatic [24].

Panitumumab, a fully humanized monoclonal antibody targeted at the EGFR, reported a 31% ORR in a phase II clinical trial by Foote and colleagues [25]. This cohort of patients only included 16 patients, with 14 patients receiving previous radiotherapy and 7 patients having received prior cytotoxic chemotherapy. Progression-free survival (PFS) was 8 months and median OS was 11 months. In a translational sub-study, total EGFR expression levels were not found to be associated with treatment efficacy.

Gefitinib and erlotinib are small TKIs molecules targeting the EGFR that have been studied in advanced cSCC. Gefitinib showed an ORR of 16% and DCR of 51% as monotherapy for advanced cSCC [26] but had a notably improved 45.5% ORR when used as neoadjuvant therapy where treatment included standard surgery or radiotherapy [27]. Erlotinib has also failed to show great efficacy as monotherapy, with a single-arm phase II trial reporting an ORR of 10% (all partial responses) although there was a notable DCR of 72% [28]. Erolitinib combined with surgery and postoperative adjuvant radiation therapy in a phase I trial showed a recurrence rate of 26.7% with mean time to recurrence in 10.5 months, again highlighting the use of these agents in combination with other systemic agents or therapeutic modalities [29]. Lapatinib, a TKI that blocks the HER2 receptor, has been found to be anecdotally effective in treatment of advanced cSCC [30], prompting larger trials to investigate its efficacy in larger cohorts [31].

EGFR-targeted therapies are typically more tolerable with adverse effects being less severe when compared to cytotoxic agents. Cutaneous adverse events are incredibly frequent, occurring from 50–100% of the time in reported clinical trials using these agents, and can be a cause of poor drug compliance and/or drug cessation [32]. Acneiform eruptions in seborrheic areas are common, but patients may also develop xerosis, paronychia, and other cutaneous toxicities. There is a positive correlation between the occurrence and severity of cutaneous adverse effects and tumor response, which can be a supporting detail to discuss with patients when helping manage these skin toxicities [33]. Due to significant EGFR expression in epithelial cells of the gastrointestinal tract, diarrhea is a common adverse event of EGFR targeted agents, with incidence varying from 27% to 87% in various phase III clinical trials for various malignancies [34]. Nevertheless, these adverse events are more manageable in comparison to the severe side effects that can occur with cytotoxic therapies.

Intrinsic and acquired resistance to EGFR inhibitors is well recognized [35]. Several mechanisms of resistance in advanced malignancies have been described and many include genomic alterations in downstream effectors of the EGFR signaling pathway [36]. These may arise either as new genetic alterations in the tumor after start of therapy, or through positive selection pressure of anti-EGFR therapies on an already-present group of EGFR-resistant cells present at time of treatment initiation [37]. Strategies to combat anti-EGFR resistance include the use of combination therapy with other therapeutic modalities such as radiotherapies, or combination therapies with other systemic agents including traditional cytotoxic chemotherapy [38].

Table 2 summarizes a small sample of studies investigating targeted therapies for treatment of advanced cSCC. Treatment with these agents may be limited due to short durations of response and progression free survival.

### Systemic Immunotherapy for Advanced cSCC

#### Immunogenicity of Cutaneous Squamous Cell Carcinoma

A discussion of the immune system’s role in development of cSCC and tumor progression is essential to understanding how immunotherapy acts and mediates destruction of target tumor cells. It is known that immunosuppression significantly increases the incidence of non-melanoma skin cancer including cSCC which occurs 65–250 times more frequently in patients after organ transplantation compared to the general population [39]. In addition, cSCC in the organ transplant population tend to be more aggressive with an increased risk of local recurrence, regional and distant metastasis, and mortality [40]. Chronic immunosuppression impairs normal immune surveillance and eradication of carcinogenic changes within tumor cells. Ultraviolet (UV) radiation, a primary pathogenic factor for development of squamous cell carcinoma, directly causes UV-specific mutations in keratinocytes as well as a functional and quantitative reduction in the cutaneous immune response, which in turn suppresses the cutaneous antigen presentation and recognition in the cutaneous environment [41]. Cutaneous SCC has one of the highest mutational burden (TMB) of all tumors including melanoma and other squamous tumor types [42]. TMB has shown to have predictive value of tumor response using immune checkpoint inhibitors in advanced malignancies [43], making cSCC an ideal candidate for immunotherapy treatment.

Tumor cells attempt to evade the immune system through a variety of mechanisms, and those cells that succeed show a loss of expression of immunogenic tumor-specific antigens [44]. These cells are admixed with a variety of infiltrating leukocytes of both innate and adaptive origin in what is called a tumor microenvironment (TME). This microenvironment includes many complex and dynamic interactions between tumor cells and the immune cells, including T-cells which constitute a major cell type found in this microenvironment [45]. Tumor cells can evade the immune system in the TME by T-cell co-stimulation through tumor cell upregulation of immune-checkpoint co-signaling proteins such as cytotoxic T-lymphocyte-associated antigen 4 (CTLA-4) and programmed death-1 (PD-1) protein. These act as brakes on the anti-tumor immune response, and have become immunotherapeutic targets in treatment of cSCC. Exploring the vast and intricate interactions between the immune response and cSCC is beyond the scope of this review. Nevertheless, a strong emphasis should be placed on recognizing that these interactions form the backbone from which modern immunotherapeutics have been designed and developed.

#### PD-1 Inhibitors

PD-1 inhibitors have become a mainstream approach for the treatment of a variety of cancers including, but not limited to, non-small cell lung cancer, metastatic melanoma, colorectal cancer, as well as advanced cSCC [46]. The PD-1 receptor protein on T-cells interacts with its corresponding ligand, PD-L1 or PD-L2, which may be expressed on tumor cells or tumor infiltrating lymphocytes that may be in the TME. This interaction transmits downstream signals that inhibit T-cell proliferation, cytokine production, and cytolytic function, thus inhibiting the immunogenic antitumor response [47–49]. PD-1 inhibitors are one type of immune checkpoint inhibitor that blocks the PD-1/PD-L1 interaction and allows for enhanced immune surveillance and destruction of tumor cells.

Pembrolizumab, nivolumab, and most recently cemiplimab are anti-PD1 monoclonal antibodies used in the treatment of advanced malignancies. In 2018, registration trials by Migden and colleagues showed ORRs of 41.1–50% in the phase I and II studies that included patients with locally advanced or metastatic cSCC treated with weight-based dosing of cemiplimab (3mg/kg q2weeks) or a fixed dose (350 mg q3weeks). Disease control rates ranged from 62–71.2% [50–52]. It is worthwhile to note that there were patients included in these trials where partial response was not obtained until after 6–10 months of treatment and thus only a few cycles of therapy seem insufficient to determine response. Some patients who initially appeared as non-responders later showed impressive tumor regression and at times a complete response. The KEYNOTE-629 trial by Grob and colleagues, a single arm phase II trial that treated 105 patients with 200 mg pembrolizumab q3 weeks (Q3W), observed a 34.4% ORR with a 52.4% DCR [53]. It should be noted that in 86.7% of patients in the KEYNOTE-629 trial, pembrolizumab was at least second line therapy. Maubec and colleagues reported a slightly higher ORR of 41% and DCR of 54% in a phase II study of pembrolizumab as first-line monotherapy [54]. Although nivolumab lacks completed large scale phase I and II trials for advanced cSCC as seen with cemiplimab and pembrolizumab, it has been used as therapy for advanced cSCC. Table 3 summarizes several studies investigating anti-PD1 therapies for advanced cSCC.

Immunotherapy brings with it a wide variety of possible immune related adverse events (irAEs) involving various organ systems that may be monitored, managed symptomatically, or treated with systemic steroids if grade 3 or higher. In the large-scale trials of cemiplimab mentioned above, the most reported side effects in the study were diarrhea and fatigue, experienced by 25% of enrolled patients, of which the majority were low grade [50]. Patients should be very closely monitored and informed to report even the mildest of symptoms, as life-threatening irAE such as colitis, pneumonitis, and hepatitis represent a possible downside of immunotherapy. Most clinical trials involving immunotherapy have excluded patients with a prior history of autoimmune disease. Those that included patients with a history of autoimmune disease showed that anti-PD1 agents may cause disease flares. Although some patients did experience significant anti-tumor activity, the degree of flares did not correlate with degree of response [55].

PD-1 inhibitors are not excluded from the shortfalls of intrinsic or acquired tumor resistance. In the open-label, multicohort phase 1b clinical trial for patients with metastatic melanoma, 25% of patients treated with PD-1 inhibitors who demonstrated an objective response later developed disease progression [56]. Mechanisms of resistance are believed to include loss of cell function, disruption of antigen presentation, as well as several other immune-associated factors [57]. Acquired resistance of PD-1 inhibitors in non-melanoma skin cancer has not been frequently reported or characterized at this time, but may be revealed as additional larger trials with these agents are completed.

Intralesional therapy of immune checkpoint inhibitors are an appealing approach to treatment. This may allow for higher local tissue concentrations of the agent within the tumor site, with limited systemic exposure and possibly lower systemic toxicity. A study investigating intralesional PD-1 inhibitor therapy for recurrent cSCC is currently underway [58].

#### PD1/PD-L1 Expression and Tumor Response

Identification of tumors that are most likely to respond to anti-PD1 agents has been difficult. Studies on PD1 inhibitors for the treatment of various advanced malignancies have shown that pretreatment tumor PD-L1 expression has a potential association with response to the PD-1 pathway blockade [59]. However, in other cases, PD-L1 negative patients showed excellent response to treatment whereas PD-L1 positive patients showed no response to therapy [60–62]. There are no established guidelines, understandably, when considering how and when to biopsy a tumor sample. Small needle biopsies versus punch biopsies of cutaneous tumors may provide differing results. In addition, gene expression is not uniform throughout a tumor [63] and 1 biopsy alone may be far from sufficient to provide adequate sampling. In some instances, PD-L1 expression is limited or may even be absent in tumor cells while significant expression of this marker was noted in the tumor infiltrating lymphocytes. Variations between methods used including immunohistochemistry and staining techniques, as well as definitions of PD-L1 ‘positivity’ (cell surface expression, cytoplasmic expression, by tumor cells only, by immune related cells, threshold of positivity) make for less conclusive predictive value of these markers. There is much more work needed to determine the validity and reliability of PD-L1 expression as a predictor of tumor response.

#### Anti-PDL1 agents

Complementing the anti-PD1 agents discussed above, anti-PDL1 agents have been developed in hopes of adding yet another agent in our fight against advanced malignancies. Atezolizumab, Avelumab, and Durvalumab are IgG1 antibodies targeting against the PD-L1 ligand. These agents have been used for treatment of various malignancies including urothelial carcinoma, non-small cell lung cancer, and metastatic Merkel-cell carcinoma. In 2017, there were 117 ongoing clinical trials that involved atezolizumab [64]. Although there are studies using anti-PDL1 agents for advanced cSCC, there is no data published at this time. The second PD-1 ligand, PD-L2, is selectively expressed on monocytes and dendritic cells and could similarly be another molecular target [65].

### RP1 Monotherapy and in Combination with PD-1 Inhibitors

Oncolytic virotherapy (OV) is the use of a replication-competent virus for the treatment of cancer. OV must be non-pathogenic to normal cells and can work in a variety of mechanisms to selectively kill tumor cells. In the last few decades, commercially available OVs have been used in a variety of cancers including metastatic melanoma [66]. OV has the ability to stimulate an anti-cancer immune response, increasing the immune activity within the TME. By enhancing this immune response, targeting tumor cells, and exposing tumor neoantigens, OV could provide a logistical complement to immune checkpoint inhibition. Unfortunately, a detailed discussion of OVs and their effects on the TME is beyond the scope of this review. An open-label, single-arm phase II clinical trial of the oncolytic HSV RP1 in combination with nivolumab has shown early promising results, with 5 of 6 metastatic cSCC patients enrolled showing disease response and 3 complete responses [67]. Larger ongoing studies will evaluate the efficacy and durability of responses in patients with advanced cSCC.

### CTLA-4 Inhibitors

Ipilimumab is a CTLA-4 blocking antibody that became the first checkpoint inhibitor to be tested and shown to be effective for the treatment of cancer patients, specifically metastatic melanoma [68]. In 2015, ipilimumab showed durable complete responses in patients with metastatic melanoma and became a standard-of-care adjuvant treatment of resected stage III melanoma [69]. Day and colleagues reported a durable response in metastatic cSCC using ipilimumab, although larger trials are needed to fully characterize ipilimumab’s safety and efficacy for this cancer [70].

The combination of ipilimumab and nivolumab has reported increased efficacy over that of single agent checkpoint blockade. These effects were more durable and significantly prolonged survival of responsive patients for various malignancies [71]. This combination therapy is currently under investigation for the treatment of advanced cSCC [72].

### Summary of Systemic Therapy for Advanced cSCC

Advanced cSCC manifests with highly variable presentation having many patient-specific features and considerations. Although some early use of cytotoxic chemotherapy agents for advanced cSCC showed impressive response rates, many of these trials included a combination of agents, as well as subsequent surgery or radiation therapy. The limitations of cytotoxic chemotherapy include their significant and at times lethal side effect profiles which is poorly tolerated in the elderly population that characterizes advanced cSCC. EGFR targeted therapies such as cetuximab showed reasonable responses, with less toxic side effect profiles that included cutaneous eruptions and gastrointestinal adverse events. However, the durability of response was suboptimal with a high percentage of tumor recurrence and low overall survival at the 2-year mark. Immunotherapy with PD-1 inhibitors demonstrate good response rates and generally good tolerability. Although severe irAEs have occurred, lower grade adverse events are more common. As monotherapy, large scale trials of PD1 inhibitors showed ORRs close to 50%. While biomarkers are conceptually well suited for deciding which systemic agents would provide the most clinical benefit, assessment of these to date has yielded mostly inconsistent results. Patients with advanced cSCC treated with cetuximab showed efficacy in a cohort of patients that did not reveal significant EGFR mutations, although this is specific to the investigated *loci* in the study by Picard and colleagues [73]. As noted above, similar findings have been noted with PD-1 inhibitors where complete responses occurred in tumors that lacked tumor and TIL PD-L1 expression, or the lack of response was seen in tumors that strongly expressed PD-L1[60–62]. In addition, differences in the methods used for biomarker testing or other assays further confound the interpretation of these results [74]. Standardization of biomarker testing in large-scale trials may lead to more conclusive findings of any correlations identified.

## Conclusions

Significant advances have been made in the quest to achieve durable tumor responses with limited systemic toxicity in the fight against advanced cSCC. Although we have reviewed numerous agents and trials including cytotoxic chemotherapies, targeted agents, and immunotherapies, head-to-head comparisons are not appropriate due to the differences in study design. There are substantial differences of the demographics enrolled in these studies, and many trials did not involve first line therapy. Although different drugs within the same class may work in a similar fashion, we cannot assume that they are equivalent in efficacy or safety. Nevertheless, the rise of immunotherapy, specifically PD-1 inhibitors, have shown adequate tumor responses with less frequent severe systemic toxicities. Cytotoxic agents and targeted based therapies should not be altogether forgotten, and can continue to be considered in specific cases where immune checkpoint inhibition is contraindicated. The large number of trials in progress today include adjuvant, neoadjuvant monotherapy and combinations of the agents discussed in this review. Even so, much work is needed to improve outcomes while decreasing risks from adverse events in the fight against advanced cSCC.

## Figures and Tables

**Table 1:** Cytotoxic Chemotherapy for the Treatment of cSCC

Study	Regimen	Line of therapy	Pts (n)	Response Rate	Duration of Response	Notes/Limitations
Guthrie T *et al*. 1985[75]	Cisplatin + doxorubicin	Mixed First/Second+	4	CR 25% PR 50% ORR 75%	3–24 months	One patient had subsequent ST
Guthrie T *et al*. 1990[14]	Cisplatin + doxorubicin	Mixed First/Second+	12	CR 33% PR 25% ORR 58%	-	Sole therapy (7) OR neoadjuvant (5) receiving RT or ST
Sadek H *et al*. 1990[13]	Cisplatin + 5-fluorouracil^†^ + Bleomycin	Second+	13	CR 30% PR 54% ORR 84%	-	Almost all pts received subsequent ST/RT
Khansur T *et al*. 1991[76]	Cisplatin + 5-fluorouracil	First line	7	CR 43% PR 43% ORR 86%	-	One patient received subsequent ST. Several patients lost to follow up
Merimsky O *et al*. 1992[77]	Adriamycin + Cisplatin	Second+ Line	2	CR 50% PR 50% ORR 100%	-	Both patients had only locally advanced disease.
Cartei G *et al*. 2000[78]	Oral 5-fluorouracil	Second+ Line	14	CR 0% PR 14% ORR 14%	Median 30 weeks	None had distant metastasis. Some received oral + topical 5-FU
Shin DM *et al*. 2002[79]	IFN-alpha and 13-cis-retinoic acid and cisplatin	Mixed First/Second+	39	CR 17% PR 17% ORR 34%	Median 9 months	None had distant metastasis. Median survival 14.6 months.
Fujisawa Y *et al*. 2006[80]	Cisplatin + 5-fluorouracil + RT	First Line	2	CR100% PR 0% ORR 100%	-	Both patients had only locally advanced disease.
Nottage MK *et al*. 2017[15]	(cisplatin OR carboplatin) + RT	First Line	19	CR 53% PR 47% ORR 100%	-	Median survival just over 24 months

Note this table is just a limited sampling of published reports including cytotoxic chemotherapy for advanced cutaneous SCC.

CR= complete response; PR = partial response; ORR = overall response rate; ST = surgical therapy; RT = radiation therapy; Pts = patients; IFN-alpha = interferon alpha

**Table 2:** Targeted Therapies for the Treatment of Advanced Cutaneous SCC

Study	Regimen	Line of Therapy	Pts (n)	Response Rate	Duration of Response	Notes/Limitations
Maubec E *et al*. 2011[23]	Cetuximab	First Line (42%) Second+ Line (58%)	36	CR 6% PR 22% ORR 28%	Mean 6.8 months	Mean OS 8.1 months Median PFS 4.1 months DCR of 69% Some patients received subsequent surgical excision
Montauedié H *et al*. 2020[24]	Cetuximab	First Line (36%) Second+ Line (64%)	58	CR 2% PR 40% ORR 42%	-	Retrospective study DCR at 12 weeks 70% Median PFS 9.7 months Median OS 17.5 months
Hillen U. *et al*. 2018[81]	Cetuximab	Majority Second+ Line	15	CR 6% PR 13% ORR 20%	Mean 7 months	Retrospective study
Preneau S *et al*. 2014[82]	Cetuximab (6), Cetuximab + RT (5), Cetuximab + Carboplatin (9)	First Line (10%) Second+ Line (90%)	20	ORR 47%	-	OS 11.1 months PFS 5.7 months ORR: Cetuximab monotherapy (33%), Cetuximab + carboplatin (38%), Cetuximab + RT (80%)
Reigneau M *et al*. 2015[83]	Neoadjuvant Cetuximab +/- (platinum based agent + 5-Fluorouracil) prior to ST	Mixed First Line and Second+ Line	34	-	-	Locally advanced SCC only 9 Pts received Cetuximab Monotherapy Tumors became resectable in 28 of 34 patients (82%) Some patients received adjuvant RT
Galbiati D *et al*. 2019[17]	Cetuximab + (Cisplatin OR Carboplatin)	Second+ Line	12	CR 8% PR 42% ORR 50%	Median 4.8 months	Median PFS 6.6 months Median OS 14.6 months Some patients subsequently received ST or RT
Foote MC *et al*. 2014[25]	Panitumumab	First Line (6%) Second+ Line (92%)	16	CR 12 % PR 19% ORR 31%	Median 5 months	Median PFS 8 months Median OS 11 months
William WN *et al*. 2017[26]	Gefitinib	Second+ Line	37	CR 0% PR 16% ORR 16%	Median 31.4 months	DCR 51% Median OS 12.9 months Median PFS 3.8 months
Lewis *et al*. 2012[27]	Neoadjuvant Gefinitib prior to ST and/or RT	First Line (43%) Second+ line (57%)	22	CR 18% PR 27% ORR 45%	-	2-year OS 72.1% 2-year PFS 63.6%
Gold *et al*. 2018[28]	Erlotinib	Second+ Line	29	CR 0% PR 10% ORR 10%	-	DCR 72% Median OS 13 months Median PFS 4.7 months

Note this table is just a limited sampling of published reports including targeted therapies for advanced cutaneous SCC.

CR= complete response; PR = partial response; ORR = overall response rate; ST = surgical therapy; RT = radiation therapy; Pts = patients; DCR = Disease Control Rate; SCC = Squamous Cell Carcinoma.

**Table 3:** Anti-PD1 Therapies for Advanced cSCC

Study	Regimen	Line of Therapy	Pts (n)	Response Rate	Duration of Response	Notes/Limitations
Migden MR *et al*. 2018 [50]	Cemiplimab	First Line (31%) Second+ Line (69%)	26	CR 0% PR 50% ORR 50%	Median NR	Expansion Cohorts of Phase I Study including Locally Advanced or Metastatic cSCC
Migden MR *et al*. 2020[52]	Cemiplimab	First and Second+ Line	78	CR 13% PR 31% ORR 44%	Median NR KM DOR 89%	Phase II Locally advanced cSCC Cohort KM 1 year OS 81% KM 1 year PFS 53%
Richscin D *et al*. 2020[51]	Cemiplimab	First and Second+ Line	71	CR 11% PR 34% ORR 45%	Median NR KM DOR 90%	DCR 68% KM 1 year OS 81% KM 1 year PFS 51.2%
In GK *et al*. 2020[84]	Cemiplimab (13) Pembrolizumab (7) Nivolumab (6)	First and second+ Line	26	CR 23% PR 19% ORR 42%	Median 7.6 months	Retrospective study Median PFS 5.4 months
Maubec E *et al*. 2020[54]	Pembrolizumab	First Line (31%) Second+ Line (69%)	57	CR 8% PR 33% ORR 41%	Median NR	DCR 60%
Salzmann M *et al*. 2020[85]	Cemiplimab (8) Pembrolizumab (28) Nivolumab (10)	First Line (67%) Second+ Line (33%)	46	CR 15% PR 44% ORR 59%	-	Retrospective study DCR 80% KM 1 year PFS 59%
Grob JJ *et al*. 2020[53]	Pembrolizumab	First Line (13%) Second+ Line (87%)	105	CR 4% PR 31% ORR 34%	Median NR	DCR 52.4% Median PFS 6.9 months Median OS NR
Eton O *et al*. 2020[86]	Pembrolizumab + panitumumab (1) Nivolumab + panitumumab (1)	Second+ Line	2	CR 100% ORR 100%		Case series

Note this table is just a limited sampling of published reports including PD-1 inhibitor therapies for advanced cutaneous SCC.

CR= complete response; PR = partial response; ORR = overall response rate; ST = surgical therapy; RT = radiation therapy; Pts = patients; DCR = Disease Control Rate; DOR= Duration of Response; cSCC = Cutaneous Squamous Cell Carcinoma; NR= Not Reached; KR = Kaplan-Meier estimate 12-month estimate
